# Whole-Body Water Flow Stimulation to the Lower Limbs Modulates Excitability of Primary Motor Cortical Regions Innervating the Hands: A Transcranial Magnetic Stimulation Study

**DOI:** 10.1371/journal.pone.0102472

**Published:** 2014-07-15

**Authors:** Daisuke Sato, Koya Yamashiro, Hideaki Onishi, Yasuhiro Baba, Sho Nakazawa, Yoshimitsu Shimoyama, Atsuo Maruyama

**Affiliations:** 1 Institute for Human Movement and Medical Sciences, Niigata University of Health and Welfare, Niigata City, Japan; 2 Department of Health and Sports, Niigata University of Health and Welfare, Niigata City, Japan; 3 Department of Physical Therapy, Niigata University of Health and Welfare, Niigata City, Japan; University of Ottawa, Canada

## Abstract

Whole-body water immersion (WI) has been reported to change sensorimotor integration. However, primary motor cortical excitability is not affected by low-intensity afferent input. Here we explored the effects of whole-body WI and water flow stimulation (WF) on corticospinal excitability and intracortical circuits. Eight healthy subjects participated in this study. We measured the amplitude of motor-evoked potentials (MEPs) produced by single transcranial magnetic stimulation (TMS) pulses and examined conditioned MEP amplitudes by paired-pulse TMS. We evaluated short-interval intracortical inhibition (SICI) and intracortical facilitation (ICF) using the paired-TMS technique before and after 15-min intervention periods. Two interventions used were whole-body WI with water flow to the lower limbs (whole-body WF) and whole-body WI without water flow to the lower limbs (whole-body WI). The experimental sequence included a baseline TMS assessment (T0), intervention for 15 min, a second TMS assessment immediately after intervention (T1), a 10 min resting period, a third TMS assessment (T2), a 10 min resting period, a fourth TMS assessment (T3), a 10 min resting period, and the final TMS assessment (T4). SICI and ICF were evaluated using a conditioning stimulus of 90% active motor threshold and a test stimulus adjusted to produce MEPs of approximately 1–1.2 mV, and were tested at intrastimulus intervals of 3 and 10 ms, respectively. Whole-body WF significantly increased MEP amplitude by single-pulse TMS and led to a decrease in SICI in the contralateral motor cortex at T1, T2 and T3. Whole-body WF also induced increased corticospinal excitability and decreased SICI. In contrast, whole-body WI did not change corticospinal excitability or intracortical circuits.

## Introduction

Many studies have investigated the utility of transcranial magnetic stimulation (TMS) to examine the effects of afferent sensory input on excitability in the human motor cortex. Motor-evoked potentials (MEPs) are affected by preceding electrical stimuli to mixed [1]–[3] or cutaneous nerves [4]–[6]. In addition, continuous afferent inputs have the capacity to alter cortical maps and modulate corticomotor excitability. Previous human studies have shown that a period of sensory stimulation increases corticomotor excitability for a period outlasting the stimulus [5], [7]. The anatomical substrate of this phenomenon is based on functionally specific reciprocal connectivity between the primary motor cortex (Brodmann area 4; MI) and primary somatosensory cortex (Brodmann areas 1, 2, and 3; SI) [8]. A physiological basis for sensory-driven enduring effects is changes in synaptic efficiency through time-dependent associative neuronal activities [9].

Various modalities of afferent input (mechanical, electrical and magnetic) have been employed in attempts to influence structures controlling motor neurons. TMS has been successfully used to map changes in motor organization, corticospinal excitability, sensorimotor organization, and intracortical circuits after periods of continuous afferent input. In many cases, the effects appear to have a somatotopical organization, in which the largest changes in MEPs are observed in the muscles nearest to the site of stimulation. Numerous studies suggest the peripheral nerve and cutaneous stimuli have no effect on F-waves [10]–[14] and that they are generally presumed to be due to an interaction at a cortical level.

For therapeutic applications, delivery of sensory stimuli to a broader area rather than a single muscle or nerve seem advantageous. We have previously shown that whole-body water immersion (WI) increases cutaneous afferent input and leads to a modulation of sensorimotor integration [15]–[17]. However, low-intensity afferent stimuli by whole-body WI did not alter corticospinal excitability or intracortical circuits. In the present study, we investigated the effect of water flow stimulation (WF) by utilizing a water flowing device. During water immersion, afferent inputs from cutaneous type II fiber groups were generated to stimulate SI, mainly the Brodmann area 3B [16]. Furthermore, during whole-body WF, the muscles and joints are forced into an unsteady motion. Thus proprioceptive inputs also activate motor cortical cells, as previously demonstrated by neurographic analyses [18]–[20]. In addition, MI excitability of the hand area is enhanced by not only vibration stimuli to the hand but also to the entire body [21]. This indicates that water flow stimulation to the whole-body might induce an increase in MI excitability of the hand.

Here, we explored the effects of whole-body WI and WF on corticospinal excitability and intracortical circuits by measuring MEP amplitudes by single-pulse TMS and conditioned MEP amplitudes using paired-pulse TMS. Our previous studies have shown that whole-body WI enhanced SEP gating [16] and modulated sensorimotor integration [17]. However, whole-body WI did not change corticospinal excitability and intracortical excitability because the afferent input during water immersion was not enough to change MI excitability [17]. Modulatory effects that outlast electrical and vibrated stimulation at different stimulus intensities is evidence that stimulus intensity can modulate motor cortical plasticity [11], [22]. Chipchase et al. [23] also suggested that stimulus intensity is an important parameter in the modulation of corticomotor excitability. Therefore, we postulated that increased excitability within the contralateral MI following whole-body WF is due to increasing stimulus intensity induced by water flow.

## Materials and Methods

### 1. Subjects and experimental design

Eight healthy, right-handed, male volunteers (age range, 19–25 years) without neurological deficits were enrolled as subjects for the present study. Written informed consent was obtained from each subject after the experimental nature of the study was fully explained. The study was conducted in accordance with the Declaration of Helsinki and approved by the local ethics committees of Niigata University of Health and Welfare.

**Figure 1:**
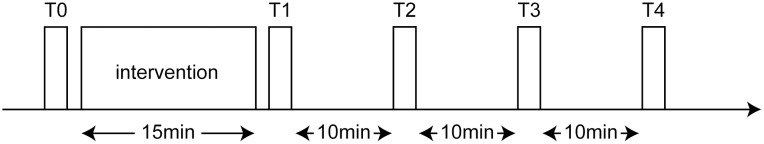
The experimental sequence used in the present study.

The subjects wore only swimwear and were seated on a comfortable reclining armchair with a mounted headrest during measurement and intervention. The experimental sequence included baseline TMS assessment (T0), intervention for 15 min, a second TMS assessment immediately after intervention (T1), a 10 min resting period, a third TMS assessment (T2), a 10 min resting period, a fourth TMS assessment (T3), a 10 min resting period, and a final TMS assessment (T4; Figure 1). This setup was performed in 3 separate protocols with 2 interventions. The two interventions were performed in a randomized order: (1) whole-body WI and (2) whole-body WF. The amplitude of the test MEP increased in response to continuous afferent stimulation in the whole-body WF trial. Therefore, we adjusted the stimulus intensity in subsequent trials so that the MEP amplitude remained the same throughout the experimental period for whole-body WF because short-interval intracortical inhibition (SICI) and intracortical facilitation (ICF) are affected by the size of the test MEP. All subjects underwent the experimental protocol with an interval between sessions of at least 5 days.

### 2. Interventions

**Figure 2:**
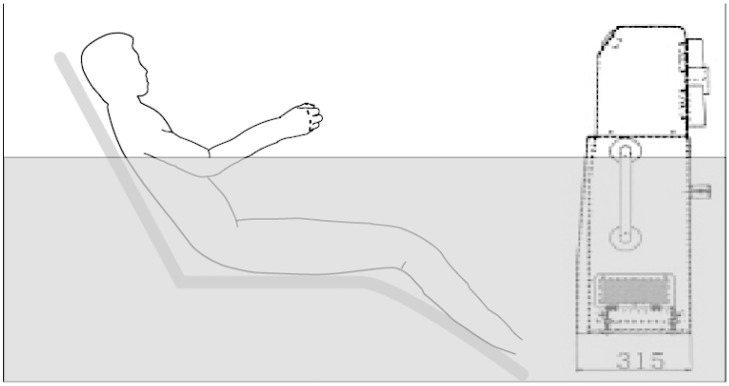
The experimental setup of the present study.

All interventions lasted for 15 min. The subjects were seated on a comfortable reclining armchair at rest on land as a control or in water for both whole-body WI and WF. The subjects assumed the same body position under all conditions using a belt to avoid muscle contractions. For whole-body WI and WF interventions, both ambient and water temperatures were set at 30°C. The tank in which the subjects were seated was filled with water up to the axillary level of the subjects. For whole-body WF intervention, the water flow stimulation was applied to the total lower limb area using a sluicing device (Flow Power-M; Japan Aqua Tech Co., Ltd, Japan). Flow velocity was set at 0.8–1.0 m/sec. Subjects were instructed to distract attention from the WF. Figure 2 shows the experimental setting of the study.

### 3. TMS assessment and electromyography (EMG) recording

TMS was performed using two Magstim 200 magnetic stimulators (Magstim Company, Ltd, Dyfed, Wales, UK) connected by a Y-cable to a figure-8 coil with an external wing diameter of 9 cm. The coil was held in place with the handle pointing backwards and laterally at approximately 45° to the sagittal plane and was optimally positioned to obtain MEPs in the right first dorsal interosseus (FDI) muscle. The site was marked on the skull to allow the experimenter to reposition the coil in the same spot before each measurement. With this coil orientation, the induced current to the brain flowed in the posterior to anterior direction.

Surface muscle responses were obtained using surface electrodes placed over the right FDI muscle using 9 mm diameter, disposable, adhesive, silver/silver-chloride surface electrodes. The active electrode was placed over the muscle belly and the reference over the interphalangeal joint of the index finger. Signals were amplified and filtered (gain×1000, 5 Hz-1 kHz; AB-601G; Nihon Kohden Corp., Tokyo, Japan) and then transferred via a micro 1401 laboratory interface (Cambridge Electronic Design, Ltd., Cambridge, UK) to a personal computer for further analysis. The magnetic stimulation threshold for eliciting responses in the FDI muscle was determined both when subjects were relaxed and during a weak background voluntary contraction of approximately 5% of the maximum value. The subjects were instructed to view the EMG activity as visual feedback to assist in complete relaxation or to maintain a constant level of background activity. The resting motor threshold (rMT) was defined as the TMS intensity required to produce responses of ≥50 µV in at least 5 of 10 successive trials. For the active motor threshold (aMT), a minimal response of 200 µV was necessary in 50% of all trials [24].

### 4. TMS paradigm

SICI and ICF were studied using the techniques described by Kujirai *et al.* [25] and Ziemann *et al.* [26]. In brief, two TMS pulses were administered through the same stimulating coil over the left motor cortex and the effect of the first (conditioning) stimulation on the second (test) stimulation was measured. The conditioning stimulus (CS) was set at an intensity of 90% AMT. The intensity of test stimulus (TS) was adjusted to elicit an unconditioned test MEP in the relaxed right FDI of approximately 1–1.2 mV peak-to-peak amplitude. The inter-stimulus intervals (ISIs) were selected as 3 or 10 ms. Each trial block consisted of 3 different stimuli; (i) test stimulus alone, (ii) tests plus conditioning stimuli at 3 ms, and (iii) test plus conditioning stimulus at 10 ms. The order of presentation of the 3 stimuli was randomized using a computer and 10 trails of each type were recorded per block. As reported previously [11], [22], [27], the amplitude of the test MEP increases in response to continuous afferent stimulation. Because SICI and ICF are affected by the size of the test MEP, we adjusted the stimulus intensity in subsequent trials so that the MEP amplitude remained the same throughout the experimental period for whole-body WF. Regarding terminology, MEP_TEST_, MEP_SICI_, and MEP_ICF_ referred to peak-to-peak amplitudes of the test MEP alone, averaged MEP in conditioned stimulus at an ISI of 3 ms, and MEP in conditioned stimulus at an ISI of 10 ms, respectively. SICI and ICF were defined as the ratios of MEP_SICI_ amplitude to MEP_TEST_ amplitude and of MEP_ICF_ amplitude to MEP_TEST_ amplitude, respectively.

### 5. Statistical analysis

For the rMT and aMT (expressed as percentage of maximum stimulator output), a 2-factorial repeated measures analysis of variance (ANOVA) was performed with the within-subject factors of “time” (5 levels: T0, T1, T2, T3, and T4) and “intervention” (2 levels: whole-body WI, and whole-body WF).

A repeated measured ANOVA was used to assess the effects of intervention on MEP amplitudes separately for each “ISI” (3 levels: single TMS, ISI 3 ms and 10 ms) with within-subject factors “time” (5 levels: T0, T1, T2, T3, and T4) and “intervention” (2 levels: whole-body WI and whole-body WF). A repeated measures ANOVA was used to assess the effect of intervention on conditioned amplitudes separately for each “ISI” (2 levels: ISI 3 ms (SICI) and 10 ms (ICF)) with within-subject factors “time” (5 levels: T0, T1, T2, T3, and T4) and “intervention” (2 levels: whole-body WI and whole-body WF).

If the assumption of sphericity was violated in Mauchly’s sphericity test, the degree of freedom was corrected using the Greenhouse–Geisser correction coefficient epsilon, and F and *p* values were recalculated. *Post-hoc* tests (Bonferroni–Dunn) were performed, and the significance level was set at 5%.

## Results

### 1. Motor threshold

**Figure 3:**
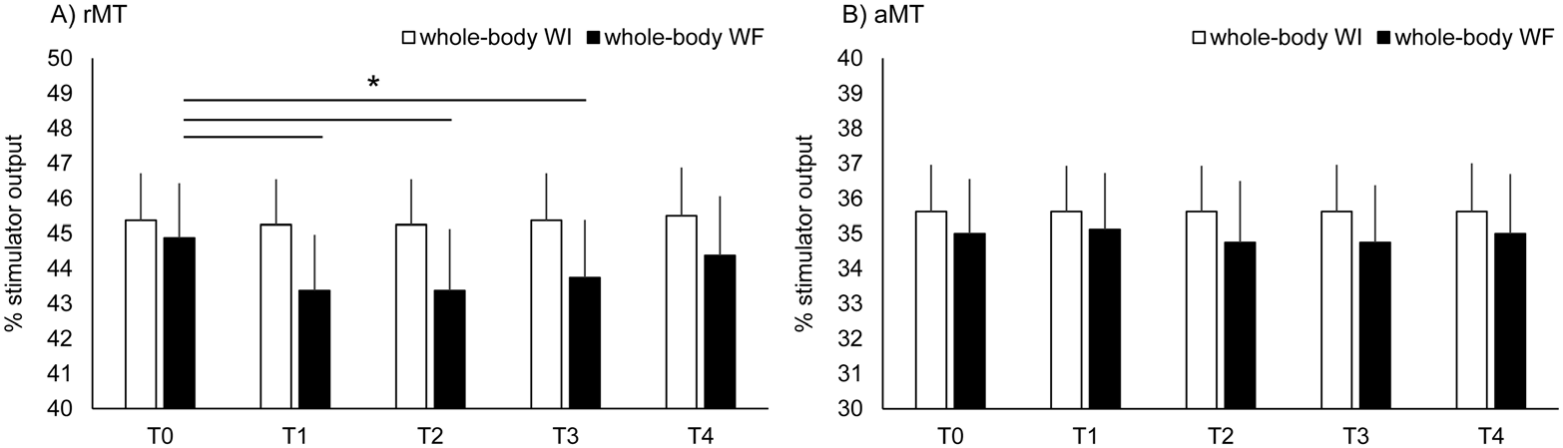
rMT and aMT for each intervention. White and black bars show the period of whole-body WI and WF, respectively. Asterisks (*) indicate significant differences (p<0.05) compared with T0.

Figure 3 presents the changes in rMT and aMT. For rMT, a repeated measures ANOVA revealed significant interactions between “intervention” and “time” (F[4], [28] = 8.01, *p*<0.01) and significant effects due to “time” (F[4], [28] = 7.52, *p*<0.01), but none of the interactions were due to “intervention” (F[1], [7] = 3.04, *p* = 0.13). *Post-hoc* comparisons at each intervention revealed significant decreases at T1, T2, and T3 compared with T0 in whole-body WF (*p*<0.05). On the other hand, for aMT, a repeated measures ANOVA revealed no interaction between “intervention” and “time” (F[4], [28] = 1.30, *p* = 0.30).

### 2. MEP amplitude induced by non-adjusted TS

**Figure 4:**
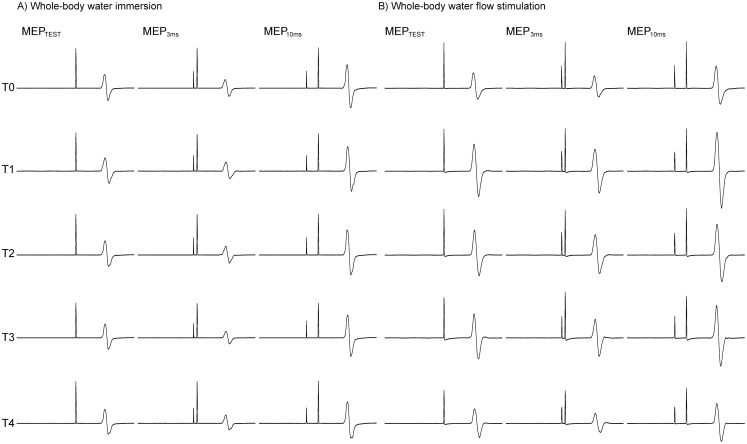
Raw data traces from a representative subject for each intervention. Averaged MEPs for the test stimulus (TS) alone, after conditioning stimulus (CS) by TMS at ISIs of 3 and 10 ms in (A) whole-body WI and (B) whole-body WF.

**Figure 5:**
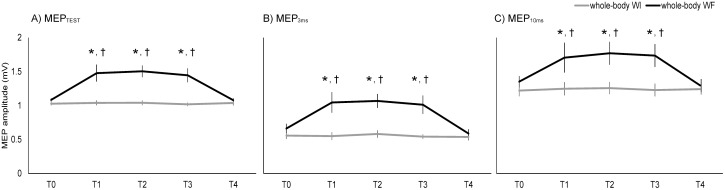
Changes in MEP amplitude for each intervention. MEP_TEST_, MEP_3 ms_, and MEP_10 ms_ were induced by the test stimulus (TS) alone, after conditioning stimulus (CS) by TMS at ISIs at 3 and 10 ms, respectively. Asterisks (*) indicate significant differences (p<0.05) compared with T0. Daggers (†) indicate significant differences (p<0.05) compared with T4.

Figures 4 and 5 present the representative waveforms and mean amplitudes of MEPs for all interventions, respectively. A repeated ANOVA revealed a significant interaction between “intervention” and “time” (F[4], [28] = 11.25, 13.49, and 5.06, *p*<0.01) for MEP_TEST_, MEP_3 ms_ and MEP_10 ms_. There were main effects of “intervention” (F[1], [7] = 22.80, *p*<0.01, F[1], [7] = 19.45, *p*<0.01, and F[1], [7] = 18.30, *p*<0.01) and “time” (F[4], [28] = 15.86, *p*<0.01, F[4], [28] = 20.03, *p*<0.01, and F[7,63] = 4.59, *p*<0.01) for MEP_TEST_, MEP_3 ms_ and MEP_10 ms_, respectively. *Post-hoc* comparisons at each ISI revealed significant increases in amplitude at T1, T2, and T3 compared with T0 and T4 in whole-body WF for MEP_TEST_ (*p*<0.05). *Post-hoc* comparisons revealed amplitudes significant increased at T1, T2, and T3 compared with T0 and T4 in whole-body WF for MEP_3 ms_ and MEP_10 ms_ (*p*<0.05).

### 3. MEP amplitude induced by adjusted TS

**Figure 6:**
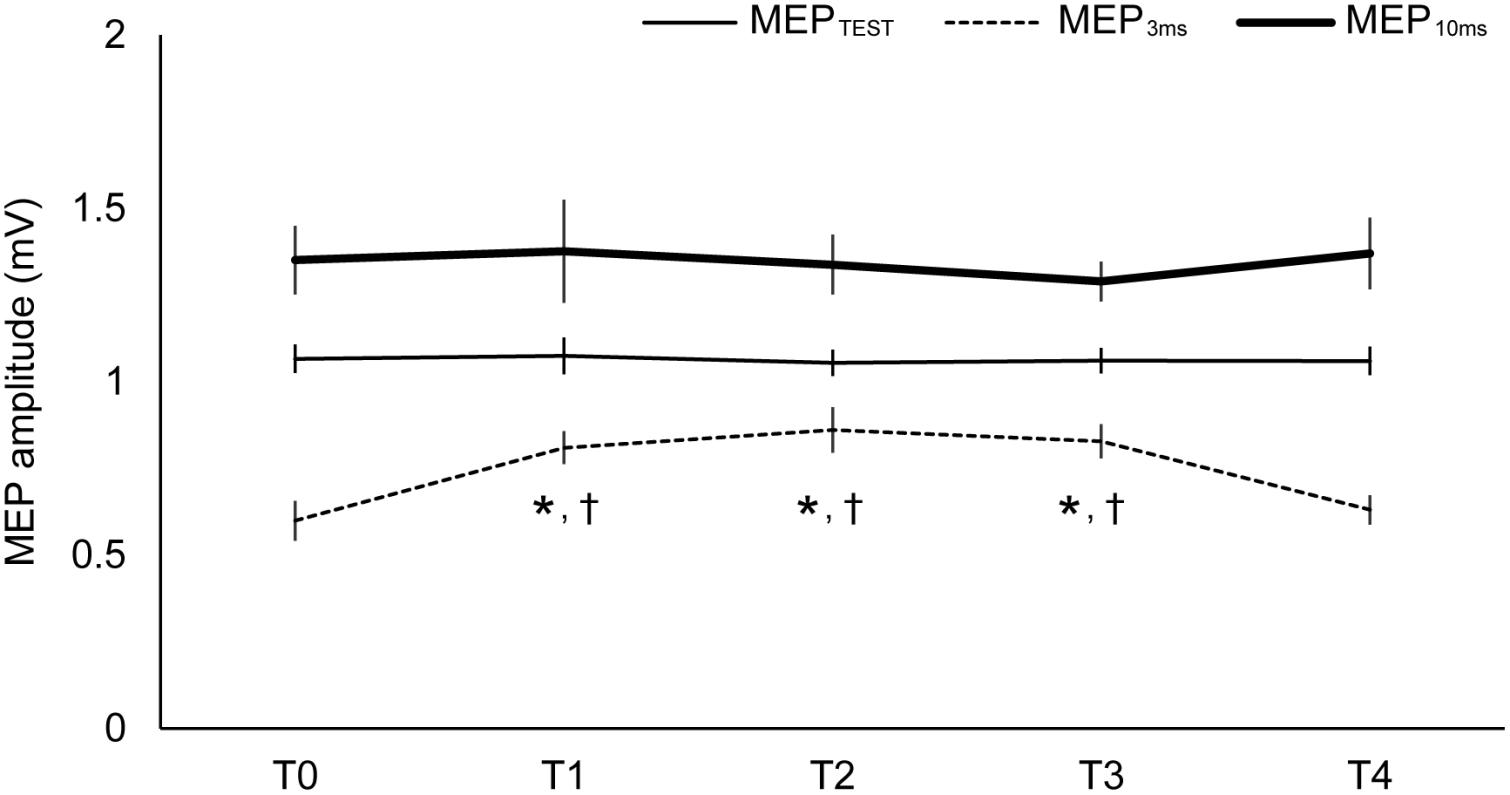
Changes in MEP amplitude induced by adjusted test stimulus (TS) in whole-body WF. TS intensity was adjusted to elicit an unconditioned test MEP in the relaxed right FDI of approximately 1 mV peak-to-peak amplitude in each assessment. Asterisks (*) indicate significant differences (p<0.05) compared with T0. Daggers (†) indicate significant differences (p<0.05) compared with T4.

Figure 6 presents the mean amplitudes of MEPs induced by adjusted TS for whole-body WF interventions. A repeated measures ANOVA revealed a significant interaction between “intervention” and “time” (F[4], [28] = 7.25, *p*<0.01) for MEP_3 ms_, but none between MEP_TEST_ (F[4], [28] = 0.20, *p* = 0.93) and MEP_10 ms_ (F[4], [28] = 0.13, *p* = 0.97). There were main effects of “time” (F[4], [28] = 7.03, *p*<0.01) for MEP_3 ms_, but not for MEP_TEST_ (F[4], [28] = 0.22, *p* = 0.93) or MEP_10 ms_ (F[4], [28] = 0.21, *p* = 0.93). *Post-hoc* comparisons revealed amplitudes significantly increased at T1, T2, and T3 compared with T0 and T4 in whole-body WF for MEP_3 m_ (*p*<0.05).

### 4. SICI and ICF

**Figure 7:**
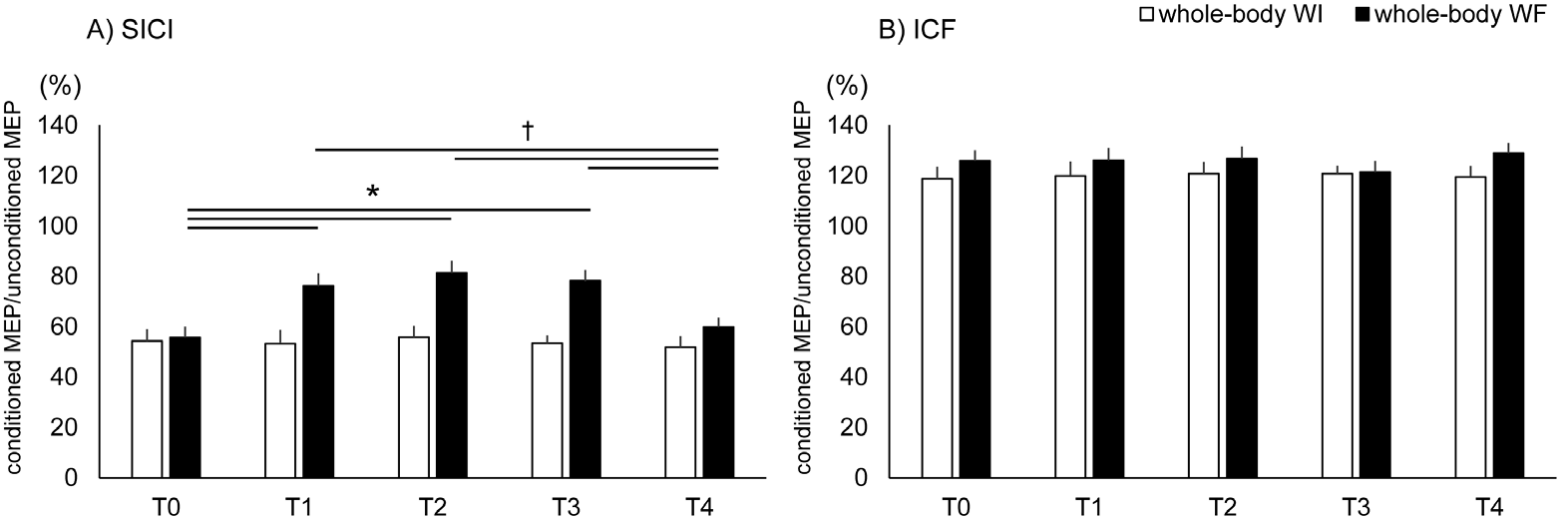
Changes in short-interval intracortical inhibition (SICI) and intracortical facilitation (ICF) for each intervention. The values for short interval intracortical inhibition and intracortical facilitation were normalized for each subject to their corresponding values in single-pulse stimulation. White and black bars show whole-body WI and WF, respectively. Asterisks (*) indicate significant differences (p<0.05) compared with T0. Daggers (†) indicate significant differences (p<0.05) compared with T4.

Figure 7 presents the changes in SICI and ICF for each intervention. A repeated measures ANOVA revealed a significant interaction “intervention” and “time” (F[4], [28] = 9.79, *p*<0.01) for SICI, but no between-interactions for ICF (F[4], [28] = 0.21, *p* = 0.93). There were main effects of “time” (F[4], [28] = 6.73, *p*<0.01) for SICI, but not for ICF (F[4], [28] = 0.17, *p* = 0.95). *Post-hoc* comparisons revealed amplitudes significantly increased at T1, T2, and T3 compared with T0 and T4 in whole-body WF for SICI (*p*<0.05).

## Discussion

In the present study, we examined the effects of whole-body WF on corticospinal excitability and intracortical inhibitory and excitatory circuits. Although only whole-body WI did not result in changes in MEP amplitude induced by single-pulse TMS, for SICI and ICF, whole-body WF increased the MEP amplitude induced by single-pulse TMS and decreased SICI. Whole-body WF did not change ICF. These results suggest that whole-body WF modulates corticospinal excitability as well as short intracortical inhibition, but not intracortical facilitation.

WI can alter numerous physiological parameters depending on physical characteristics, such as hydrostatic pressure and temperature. Several studies have revealed that WI can provide relief from edema and improve blood flow [28]–[30]. Recent studies have shown that whole-body WI can activate a large area of the somatosensory cortex and that it impacts multimodal sensory processing [16], as well as changes in sensorimotor integration [17]. The present results indicate that whole-body WI did not alter corticospinal excitability or intracortical circuits, in accordance with our previous results [17]. On the other hand, whole-body WF increased corticospinal excitability and decreased SICI, which continued for at least 30 min. The present study is the first to investigate corticospinal excitability and intracortical excitability induced by whole-body WF, and the results elucidate many neurophysiological changes that occur in the primary motor cortex. A greater understanding of these changes may allow for more effective use of aquatic therapy for neurorehabilitation.

rMT is thought to reflect neuronal membrane excitability because it is increased by drugs that block voltage-gated sodium channels [31], but not by those influencing neuronal synaptic transmission. Compared with rMT, the MEP recruitment curve was used to assess neurons that are intrinsically less excitable or are spatially further from the center of activation by TMS [32]. Therefore, the decrease in rMT and the increase in MEP_TEST_ observed in the present study indicate increased membrane excitability and stronger neural synaptic transactions.

Because the paired-pulse technique gives access to the motor cortex independently of spinal or peripheral mechanisms, it also allows for the evaluation of the intracortical circuits. There is good evidence that the interaction between a sub-threshold conditioning stimulus and a supra-threshold test stimulus at a short ISI (1–5 ms) relies on the activation of γ-aminobutyric acid (GABA), particularly GABA-A, circuits in the motor cortex [31], [33], [34]. The circuit underlying intracortical facilitation is less well understood and is thought to be mediated by glutamate [35]. Moreover, the downregulation of inhibitory neural circuits also seems to play a critical role in strengthening excitatory synapses [18]. Our findings suggest that whole-body WF to the lower limbs has a direct effect on the excitability of the intracortical circuit responsible for SICI at the cortical level. The increase in motor cortical excitability appeared to be in response to interactions in the sensorimotor cortex induced by afferent input of WF stimulation, not by water immersion.

We were uncertain as to why MI excitability increased and SICI decreased under whole-body WF at T1, T2, and T3, because the hands of the subjects were not actually immersed in the water. Rosenkranz and Rothwell [13] explored the pattern of effects on MEPs and SICI in 3 different intrinsic hand muscles after vibration of each in turn. They showed that low-amplitude vibration of a muscle can increase the amplitude of MEPs evoked in that muscle, while at the same time decrease the effectiveness of SICI and these effects exhibit a differential distribution in the vibrated versus non-vibrated hand muscles. They conclude that vibratory stimulation can produce differential change in the excitability of populations of cortical inhibitory neurons that project to different output zones of the motor cortex. However, whole-body WF in the present study differed from that in a study by Rosenkranz and Rothwell [13] with respect to the wide receptive field of the afferent input. Takahashi *et al.* [36] examined whether effects of muscle fatigue are always localized to the exercised muscle groups or to homologous muscles on the opposing side of the body, or whether more widespread effects occur if the exercise is particularly strenuous or involves very large muscle groups. The results of their study indicate that muscle fatigue induced by strenuously exercised lower limbs exhibits a decrease in excitability of the corticospinal tract and a disinhibition of intracortical circuits during the recovery period in the non-exercised upper limb. Thus afferent inputs from muscle spindles and skin in a wide receptive field, such as affected by whole-body WF, has wide-spread effects on various areas of MI, even without exercise, as we observed in the present study. After whole-body WF, effects last for 20–30 min in FDI and suggest a somatotopic spread from the lower limbs to the hand. However it is not clear what process may transmit signals along to the cortex. Another explanation for these effects might involve the connection between the MI and the posterior parietal cortex (PPC). Naito *et al.* [37] measured brain activities during tendon and skin vibration of the upper and lower limbs using functional MRI, and demonstrated that vibration of any limb activated the inferior parietal lobule (IPL), inferior frontal cortex (IF) and basal ganglia (BG). Thus, the authors indicate that right IPL activity seems to be a typical activation elicited by the sensory processing of peripheral kinesthetic inputs [37]. In addition, a twin coil transcranial magnetic stimulation approach demonstrated that PPC exerts both facilitatory and inhibitory effects towards the contralateral MI [38]. Based on these results, we propose that WF stimulation to the lower limbs can increase MI excitability and decrease SICI in the hand area via activities in PPC.

We were also uncertain as to why both whole-body WI and WF were insufficient to alter ICF because continuous afferent input induced not only an increase in coritcospinal excitability and decreased SICI but also increased ICF [22], [27]. As mentioned above, SICI and ICF are modulated by different neural mechanisms. SICI is likely because of the inhibitory effects of GABAergic interneurons. The GABAergic neurons of the cerebral cortex are aspiny nonpyramidal neurons that constitute 25%–30% of cortical neurons [19], [39]. In the motor cortex (area 4), layer II has the highest concentration of GABAergic neurons [19] with prominent vertical GABAergic projections [40]. Cortical pyramidal cells receive extensive GABAergic synapses [19]. ICF may occur because of the activation of corticocortical pyramidal cells and associated axons because they have excitatory glutaminergic synapses [39], [40]. These cells are mainly located in layers II and IIIA, and there are intracortical connections between layers III and V [39]–[42]. Labeling studies reveal extensive, long (≤3 mm), horizontally oriented intrinsic axons of pyramidal cells within the motor cortex of monkeys [40], [43]. The pattern and extent of intrinsic connections are similar throughout the motor cortex, irrespective of topographical representations [42]. In fact, vibration stimulation increases MEP_TEST_ and decreases SICI, but has no effect on ICF in the vibrated muscle because of different neural mechanisms [13]. Another possible mechanism for different effects on SICI and ICF may be related to the activation of monoamines. Ziemann *et al.* [44] assessed the modulating effects of dextroamphetamine on the excitability and stimulation-induced plasticity in the human cortex and found an increase in MEP_TEST_ amplitudes and a decrease in SICI, but no change in ICF by a specific serotonin reuptake inhibitor [45]. Water immersion induces increased vagal activity and leads to the enhanced modulation of sympathetic and parasympathetic nerve activity [46]. Therefore, further studies are required becasue the results of the present study do not directly identify the involvement of monoamine metabolism.

## Conclusion

Here, we demonstrated that whole-body WF induced increased corticospinal excitability and decreased SICI up to at least 30 min after intervention. In contrast, whole-body WI did not induce changes in corticospinal excitability of intracortical circuits.

## References

[1] Bertolasi L, Priori A, Tinazzi M, Bertasi V, Rothwell JC (1998) Inhibitory action of forearm flexor muscle afferents on corticospinal outputs to antagonist muscles in humans. J Physiol 511 (Pt 3): 947–956. 10.1111/j.1469-7793.1998.947bg.x PMC2231145 9714872

[2] Deuschl G, Michels R, Berardelli A, Schenck E, Inghilleri M, et al (1991) Effects of electric and magnetic transcranial stimulation on long latency reflexes. Exp Brain Res 83: 403–410. 2022246 10.1007/BF00231165

[3] Tokimura H, Di Lazzaro V, Tokimura Y, Oliviero A, Profice P, et al (2000) Short latency inhibition of human hand motor cortex by somatosensory input from the hand. J Physiol 523 Pt 2: 503–513. 10.1111/j.1469-7793.2000.t01-1-00503.x PMC2269813 10699092

[4] Maertens de Noordhout A, Rothwell JC, Day BL, Dressler D, Nakashima K, et al (1992) Effect of digital nerve stimuli on responses to electrical or magnetic stimulation of the human brain. J Physiol 447: 535–548. 1593458 10.1113/jphysiol.1992.sp019016 PMC1176050

[5] Ridding MC, Taylor JL (2001) Mechanisms of motor-evoked potential facilitation following prolonged dual peripheral and central stimulation in humans. J Physiol 537: 623–631. 11731592 10.1111/j.1469-7793.2001.00623.x PMC2278976

[6] Rossini PM, Tecchio F, Sabato A, Finazzi-Agro A, Pasqualetti P, et al (1996) The role of cutaneous inputs during magnetic transcranial stimulation. Muscle Nerve 19: 1302–1309. 8808656 10.1002/(SICI)1097-4598(199610)19:10<1302::AID-MUS7>3.0.CO;2-I

[7] Kaelin-Lang A, Luft AR, Sawaki L, Burstein AH, Sohn YH, et al (2002) Modulation of human corticomotor excitability by somatosensory input. J Physiol 540: 623–633. 11956348 10.1113/jphysiol.2001.012801 PMC2290238

[8] Rocco MM, Brumberg JC (2007) The sensorimotor slice. J Neurosci Methods 162: 139–147. 17307257 10.1016/j.jneumeth.2007.01.002

[9] Feldman DE (2000) Timing-based LTP and LTD at vertical inputs to layer II/III pyramidal cells in rat barrel cortex. Neuron 27: 45–56. 10939330 10.1016/s0896-6273(00)00008-8

[10] Chen R, Corwell B, Hallett M (1999) Modulation of motor cortex excitability by median nerve and digit stimulation. Exp Brain Res 129: 77–86. 10550505 10.1007/s002210050938

[11] Christova M, Rafolt D, Golaszewski S, Gallasch E (2011) Outlasting corticomotor excitability changes induced by 25 Hz whole-hand mechanical stimulation. Eur J Appl Physiol 111: 3051–3059. 21455615 10.1007/s00421-011-1933-0

[12] Classen J, Steinfelder B, Liepert J, Stefan K, Celnik P, et al (2000) Cutaneomotor integration in humans is somatotopically organized at various levels of the nervous system and is task dependent. Exp Brain Res 130: 48–59. 10638440 10.1007/s002210050005

[13] Rosenkranz K, Rothwell JC (2003) Differential effect of muscle vibration on intracortical inhibitory circuits in humans. J Physiol 551: 649–660. 12821723 10.1113/jphysiol.2003.043752 PMC2343209

[14] Tamburin S, Manganotti P, Zanette G, Fiaschi A (2001) Cutaneomotor integration in human hand motor areas: somatotopic effect and interaction of afferents. Exp Brain Res 141: 232–241. 11713634 10.1007/s002210100859

[15] Sato D, Onishi H, Yamashiro K, Iwabe T, Shimoyama Y, et al (2012) Water immersion to the femur level affects cerebral cortical activity in humans: functional near-infrared spectroscopy study. Brain Topogr 25: 220–227. 22193361 10.1007/s10548-011-0204-z

[16] Sato D, Yamashiro K, Onishi H, Shimoyama Y, Yoshida T, et al (2012) The effect of water immersion on short-latency somatosensory evoked potentials in human. BMC Neurosci 13: 13. 22272934 10.1186/1471-2202-13-13 PMC3294244

[17] Sato D, Yamashiro K, Yoshida T, Onishi H, Shimoyama Y, et al.. (2013) Effects of water immersion on short- and long-latency afferent inhibition, short-interval intracortical inhibition, and intracortical facilitation. Clin Neurophysiol. 10.1016/j.clinph.2013.04.008 23688919

[18] Hess G, Donoghue JP (1994) Long-term potentiation of horizontal connections provides a mechanism to reorganize cortical motor maps. J Neurophysiol 71: 2543–2547. 7931533 10.1152/jn.1994.71.6.2543

[19] Jones EG (1993) GABAergic neurons and their role in cortical plasticity in primates. Cereb Cortex 3: 361–372. 8260806 10.1093/cercor/3.5.361-a

[20] Jones EG, Porter R (1980) What is area 3a? Brain Res 203: 1–43. 6994855 10.1016/0165-0173(80)90002-8

[21] Mileva KN, Bowtell JL, Kossev AR (2009) Effects of low-frequency whole-body vibration on motor-evoked potentials in healthy men. Exp Physiol 94: 103–116. 18658234 10.1113/expphysiol.2008.042689

[22] Golaszewski SM, Bergmann J, Christova M, Kunz AB, Kronbichler M, et al (2012) Modulation of motor cortex excitability by different levels of whole-hand afferent electrical stimulation. Clin Neurophysiol 123: 193–199. 21764634 10.1016/j.clinph.2011.06.010

[23] Chipchase LS, Schabrun SM, Hodges PW (2011) Peripheral electrical stimulation to induce cortical plasticity: a systematic review of stimulus parameters. Clin Neurophysiol 122: 456–463. 20739217 10.1016/j.clinph.2010.07.025

[24] Ridding MC, Taylor JL, Rothwell JC (1995) The effect of voluntary contraction on cortico-cortical inhibition in human motor cortex. J Physiol 487 (Pt 2): 541–548. 10.1113/jphysiol.1995.sp020898 PMC1156591 8558482

[25] Kujirai T, Caramia MD, Rothwell JC, Day BL, Thompson PD, et al (1993) Corticocortical inhibition in human motor cortex. J Physiol 471: 501–519. 8120818 10.1113/jphysiol.1993.sp019912 PMC1143973

[26] Ziemann U, Rothwell JC, Ridding MC (1996) Interaction between intracortical inhibition and facilitation in human motor cortex. J Physiol 496 (Pt 3): 873–881. 10.1113/jphysiol.1996.sp021734 PMC1160871 8930851

[27] Golaszewski SM, Bergmann J, Christova M, Nardone R, Kronbichler M, et al (2010) Increased motor cortical excitability after whole-hand electrical stimulation: a TMS study. Clin Neurophysiol 121: 248–254. 20036618 10.1016/j.clinph.2009.09.024

[28] Bressel E, Dolny DG, Gibbons M (2011) Trunk Muscle Activity duringExercises Performed on Land and in Water. Med Sci Sports Exerc. 10.1249/MSS.0b013e318219dae7 21448084

[29] IO WM, Lepar GS, Morrissey MC, Cywinski JK (2003) Effect of neuromuscular electrical stimulation on foot/ankle volume during standing. Med Sci Sports Exerc 35: 630–634. 12673147 10.1249/01.MSS.0000058432.29149.08

[30] Wilcock IM, Cronin JB, Hing WA (2006) Physiological response to water immersion: a method for sport recovery? Sports Med 36: 747–765. 16937951 10.2165/00007256-200636090-00003

[31] Ziemann U, Lonnecker S, Steinhoff BJ, Paulus W (1996) Effects of antiepileptic drugs on motor cortex excitability in humans: a transcranial magnetic stimulation study. Ann Neurol 40: 367–378. 8797526 10.1002/ana.410400306

[32] Hallett M (1999) Motor cortex plasticity. Electroencephalogr Clin Neurophysiol Suppl 50: 85–91. 10689449

[33] Ziemann U, Lonnecker S, Steinhoff BJ, Paulus W (1996) The effect of lorazepam on the motor cortical excitability in man. Exp Brain Res 109: 127–135. 8740215 10.1007/BF00228633

[34] Hanajima R, Ugawa Y, Terao Y, Sakai K, Furubayashi T, et al (1998) Paired-pulse magnetic stimulation of the human motor cortex: differences among I waves. J Physiol 509 (Pt 2): 607–618. 10.1111/j.1469-7793.1998.607bn.x PMC2230978 9575308

[35] Liepert J, Schwenkreis P, Tegenthoff M, Malin JP (1997) The glutamate antagonist riluzole suppresses intracortical facilitation. J Neural Transm 104: 1207–1214. 9503266 10.1007/BF01294721

[36] Takahashi K, Maruyama A, Hirakoba K, Maeda M, Etoh S, et al (2011) Fatiguing intermittent lower limb exercise influences corticospinal and corticocortical excitability in the nonexercised upper limb. Brain Stimul 4: 90–96. 21511209 10.1016/j.brs.2010.07.001

[37] Naito E, Nakashima T, Kito T, Aramaki Y, Okada T, et al (2007) Human limb-specific and non-limb-specific brain representations during kinesthetic illusory movements of the upper and lower extremities. Eur J Neurosci 25: 3476–3487. 17553017 10.1111/j.1460-9568.2007.05587.x

[38] Koch G, Ruge D, Cheeran B, Fernandez Del Olmo M, Pecchioli C, et al (2009) TMS activation of interhemispheric pathways between the posterior parietal cortex and the contralateral motor cortex. J Physiol 587: 4281–4292. 19622612 10.1113/jphysiol.2009.174086 PMC2754365

[39] White EL, Keller A (1989) Cortical circuits: synaptic organization of the cerebral cortex–structure, function, and theory. Boston: Birkhäuser. xvi, 223 p.

[40] Keller A (1993) Intrinsic synaptic organization of the motor cortex. Cereb Cortex 3: 430–441. 8260811 10.1093/cercor/3.5.430

[41] Asanuma H, Rosen I (1973) Spread of mono- and polysynaptic connections within cat's motor cortex. Exp Brain Res 16: 507–520. 4695779 10.1007/BF00234477

[42] Gatter KC, Sloper JJ, Powell TP (1978) The intrinsic connections of the cortex of area 4 of the monkey. Brain 101: 513–541. 101281 10.1093/brain/101.3.513

[43] Huntley GW, Jones EG (1991) Relationship of intrinsic connections to forelimb movement representations in monkey motor cortex: a correlative anatomic and physiological study. J Neurophysiol 66: 390–413. 1723093 10.1152/jn.1991.66.2.390

[44] Ziemann U, Tam A, Butefisch C, Cohen LG (2002) Dual modulating effects of amphetamine on neuronal excitability and stimulation-induced plasticity in human motor cortex. Clin Neurophysiol 113: 1308–1315. 12140012 10.1016/s1388-2457(02)00171-2

[45] Ilic TV, Korchounov A, Ziemann U (2002) Complex modulation of human motor cortex excitability by the specific serotonin re-uptake inhibitor sertraline. Neurosci Lett 319: 116–120. 11825684 10.1016/s0304-3940(01)02563-0

[46] Perini R, Veicsteinas A (2003) Heart rate variability and autonomic activity at rest and during exercise in various physiological conditions. Eur J Appl Physiol 90: 317–325. 13680241 10.1007/s00421-003-0953-9

